# Supplementary material for: Immunological Misfiring and Sex Differences/Similarities in Early COVID-19 Studies: Missed Opportunities of Making a Real IMPACT
Source: Cells. 2023 Nov 8;12(22):2591. doi: 10.3390/cells12222591 (PMC10670326; doi:10.3390/cells12222591)
Supplement: Supplementary file 1 [file cells-12-02591-s001.zip › cells-2638127-supplementary/SupplemetaryDataCells-Revised/CellsIMPACTSupplemtalFigOct31_AB-JK.pdf]

Fig. S1

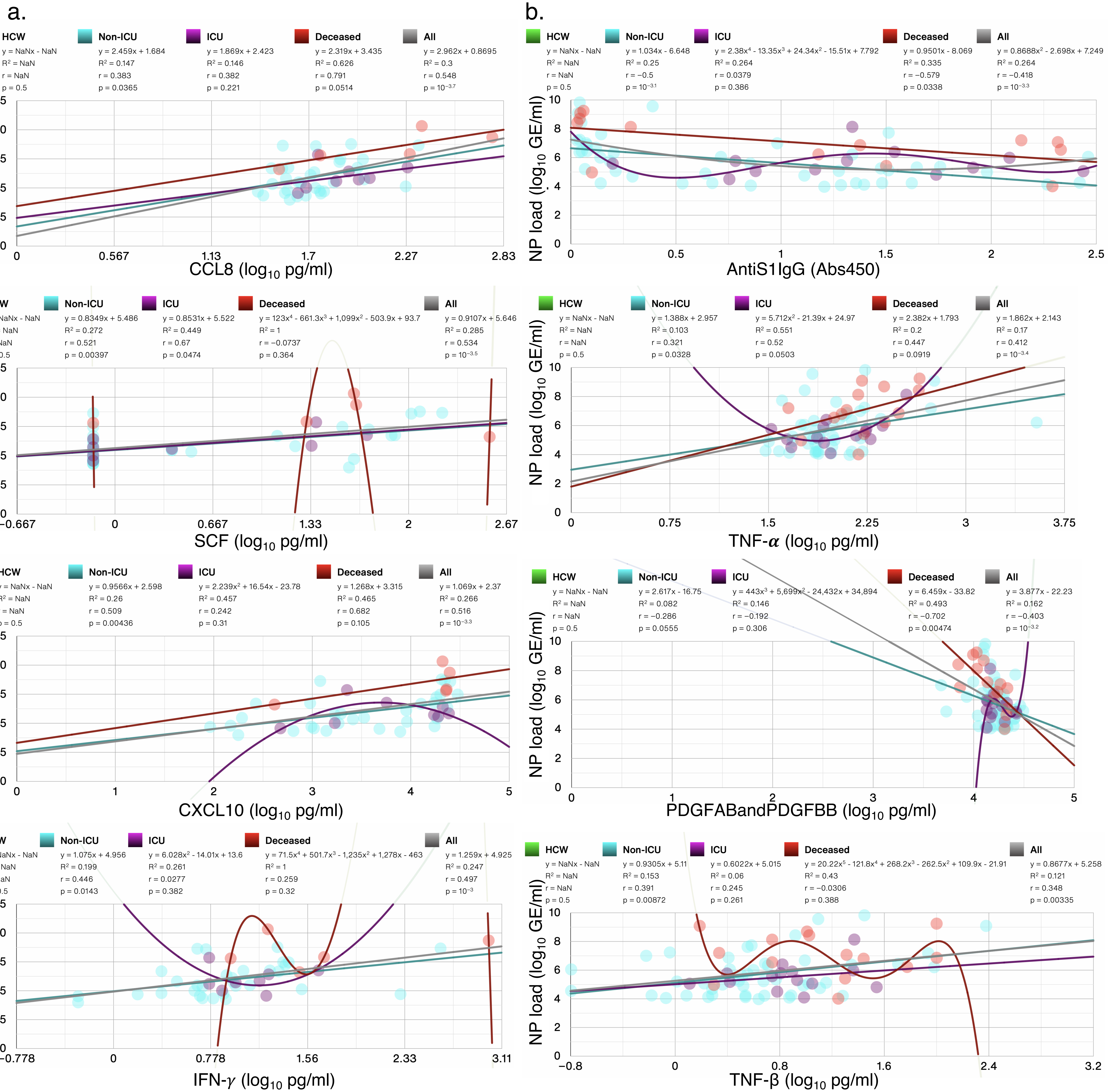
a.
HCW
y = NaNx - NaN
R² = NaN
r = NaN
p = 0.5
Non-ICU
y = 2.459x + 1.684
R² = 0.147
r = 0.383
p = 0.0365
ICU
y = 1.869x + 2.423
R² = 0.146
r = 0.382
p = 0.221
Deceased
y = 2.319x + 3.435
R² = 0.626
r = 0.791
p = 0.0514
All
y = 2.962x + 0.8695
R² = 0.3
r = 0.548
p = 10⁻³·⁷
Saliva load (log10 GE/ml)
CCL8 (log10 pg/ml)
HCW
y = NaNx - NaN
R² = NaN
r = NaN
p = 0.5
Non-ICU
y = 0.8349x + 5.486
R² = 0.272
r = 0.521
p = 0.00397
ICU
y = 0.8531x + 5.522
R² = 0.449
r = 0.67
p = 0.0474
Deceased
y = 123x⁴ - 661.3x³ + 1,099x² - 503.9x + 93.7
R² = 1
r = −0.0737
p = 0.364
All
y = 0.9107x + 5.646
R² = 0.285
r = 0.534
p = 10⁻³·⁵
Saliva load (log10 GE/ml)
SCF (log10 pg/ml)
HCW
y = NaNx - NaN
R² = NaN
r = NaN
p = 0.5
Non-ICU
y = 0.9566x + 2.598
R² = 0.26
r = 0.509
p = 0.00436
ICU
y = 2.239x² + 16.54x - 23.78
R² = 0.457
r = 0.242
p = 0.31
Deceased
y = 1.268x + 3.315
R² = 0.465
r = 0.682
p = 0.105
All
y = 1.069x + 2.37
R² = 0.266
r = 0.516
p = 10⁻³·³
Saliva load (log10 GE/ml)
CXCL10 (log10 pg/ml)
HCW
y = NaNx - NaN
R² = NaN
r = NaN
p = 0.5
Non-ICU
y = 1.075x + 4.956
R² = 0.199
r = 0.446
p = 0.0143
ICU
y = 6.028x² - 14.01x + 13.6
R² = 0.261
r = 0.0277
p = 0.382
Deceased
y = 71.5x⁴ + 501.7x³ - 1,235x² + 1,278x - 463
R² = 1
r = 0.259
p = 0.32
All
y = 1.259x + 4.925
R² = 0.247
r = 0.497
p = 10⁻³
Saliva load (log10 GE/ml)
IFN-γ (log10 pg/ml)
b.
HCW
y = NaNx - NaN
R² = NaN
r = NaN
p = 0.5
Non-ICU
y = 1.034x - 6.648
R² = 0.25
r = −0.5
p = 10⁻³·¹
ICU
y = 2.38x⁴ - 13.35x³ + 24.34x² - 15.51x + 7.792
R² = 0.264
r = 0.0379
p = 0.386
Deceased
y = 0.9501x - 8.069
R² = 0.335
r = −0.579
p = 0.0338
All
y = 0.8688x² - 2.698x + 7.249
R² = 0.264
r = −0.418
p = 10⁻³·³
NP load (log10 GE/ml)
AntiS1IgG (Abs450)
HCW
y = NaNx - NaN
R² = NaN
r = NaN
p = 0.5
Non-ICU
y = 1.388x + 2.957
R² = 0.103
r = 0.321
p = 0.0328
ICU
y = 5.712x² - 21.39x + 24.97
R² = 0.551
r = 0.52
p = 0.0503
Deceased
y = 2.382x + 1.793
R² = 0.2
r = 0.447
p = 0.0919
All
y = 1.862x + 2.143
R² = 0.17
r = 0.412
p = 10⁻³·⁴
NP load (log10 GE/ml)
TNF-α (log10 pg/ml)
HCW
y = NaNx - NaN
R² = NaN
r = NaN
p = 0.5
Non-ICU
y = 2.617x - 16.75
R² = 0.082
r = −0.286
p = 0.0555
ICU
y = 443x³ + 5,699x² - 24,432x + 34,894
R² = 0.146
r = −0.192
p = 0.306
Deceased
y = 6.459x - 33.82
R² = 0.493
r = −0.702
p = 0.00474
All
y = 3.877x - 22.23
R² = 0.162
r = −0.403
p = 10⁻³·²
NP load (log10 GE/ml)
PDGFABandPDGFBB (log10 pg/ml)
HCW
y = NaNx - NaN
R² = NaN
r = NaN
p = 0.5
Non-ICU
y = 0.9305x + 5.11
R² = 0.153
r = 0.391
p = 0.00872
ICU
y = 0.6022x + 5.015
R² = 0.06
r = 0.245
p = 0.261
Deceased
y = 20.22x⁵ - 121.8x⁴ + 268.2x³ - 262.5x² + 109.9x - 21.91
R² = 0.43
r = −0.0306
p = 0.388
All
y = 0.8677x + 5.258
R² = 0.121
r = 0.348
p = 0.00335
NP load (log10 GE/ml)
TNF-β (log10 pg/ml)

Fig.S2

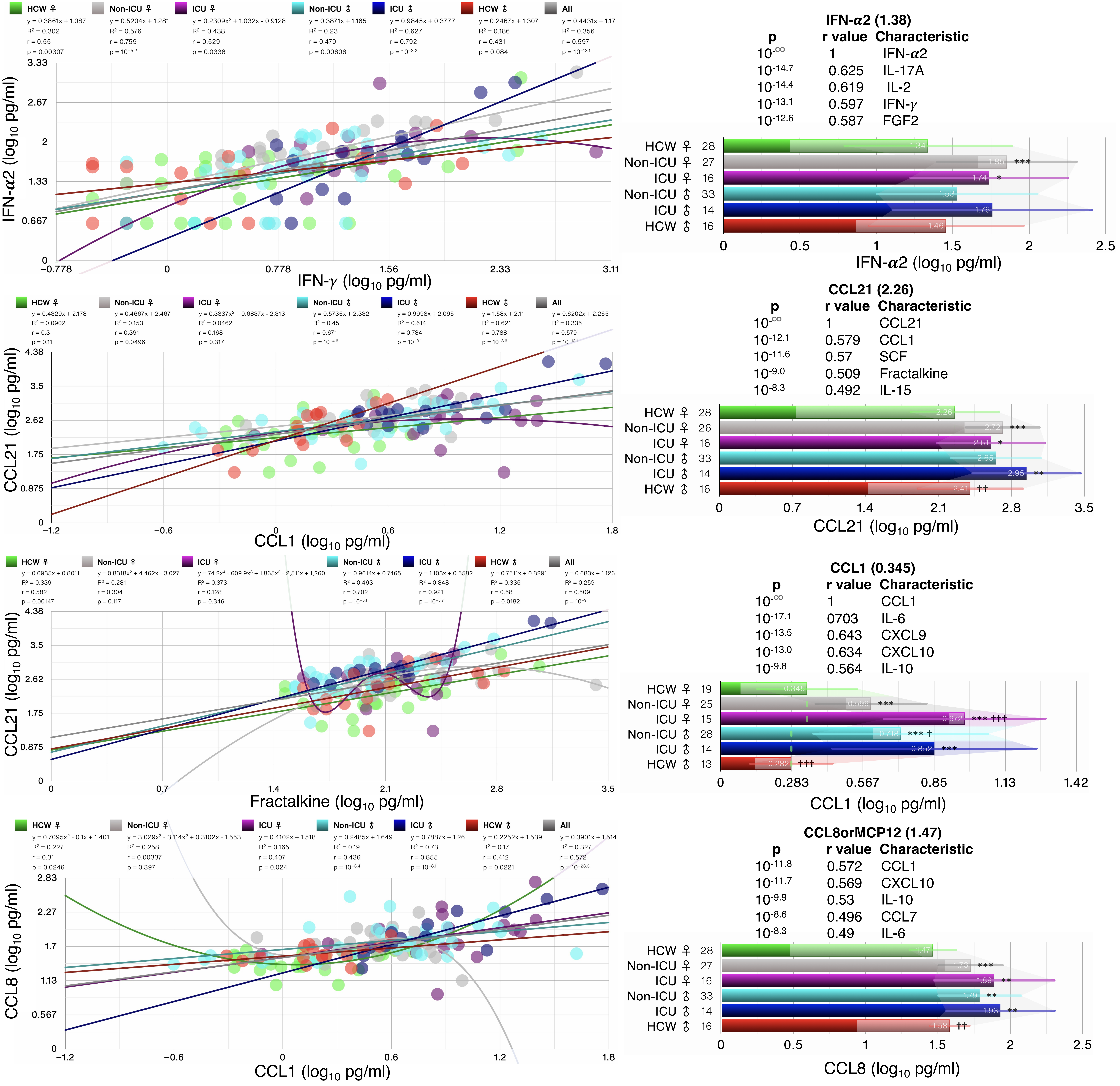

HCW ♀
Non-ICU ♀
ICU ♀
Non-ICU ♂
ICU ♂
HCW ♂
All
y = 0.3861x + 1.087
R² = 0.302
r = 0.55
p = 0.00307
y = 0.5204x + 1.281
R² = 0.576
r = 0.759
y = 0.2309x² + 1.032x - 0.9128
R² = 0.438
r = 0.529
p = 0.0336
y = 0.3871x + 1.165
R² = 0.23
r = 0.479
p = 0.00606
y = 0.9845x + 0.3777
R² = 0.627
r = 0.792
y = 0.2467x + 1.307
R² = 0.186
r = 0.431
p = 0.084
y = 0.4431x + 1.17
R² = 0.356
r = 0.597
IFN-α2 (log10 pg/ml)
IFN-γ (log10 pg/ml)
IFN-α2 (1.38)
p
r value
Characteristic
1
IFN-α2
0.625
IL-17A
0.619
IL-2
0.597
IFN-γ
0.587
FGF2
CCL21 (log10 pg/ml)
CCL1 (log10 pg/ml)
CCL21 (2.26)
0.579
CCL1
0.57
SCF
0.509
Fractalkine
0.492
IL-15
Fractalkine (log10 pg/ml)
CCL1 (0.345)
0703
IL-6
0.643
CXCL9
0.634
CXCL10
0.564
IL-10
CCL8 (log10 pg/ml)
CCL8orMCP12 (1.47)
0.572
CCL1
0.569
CXCL10
0.53
IL-10
0.496
CCL7
0.49
IL-6

Fig. S3

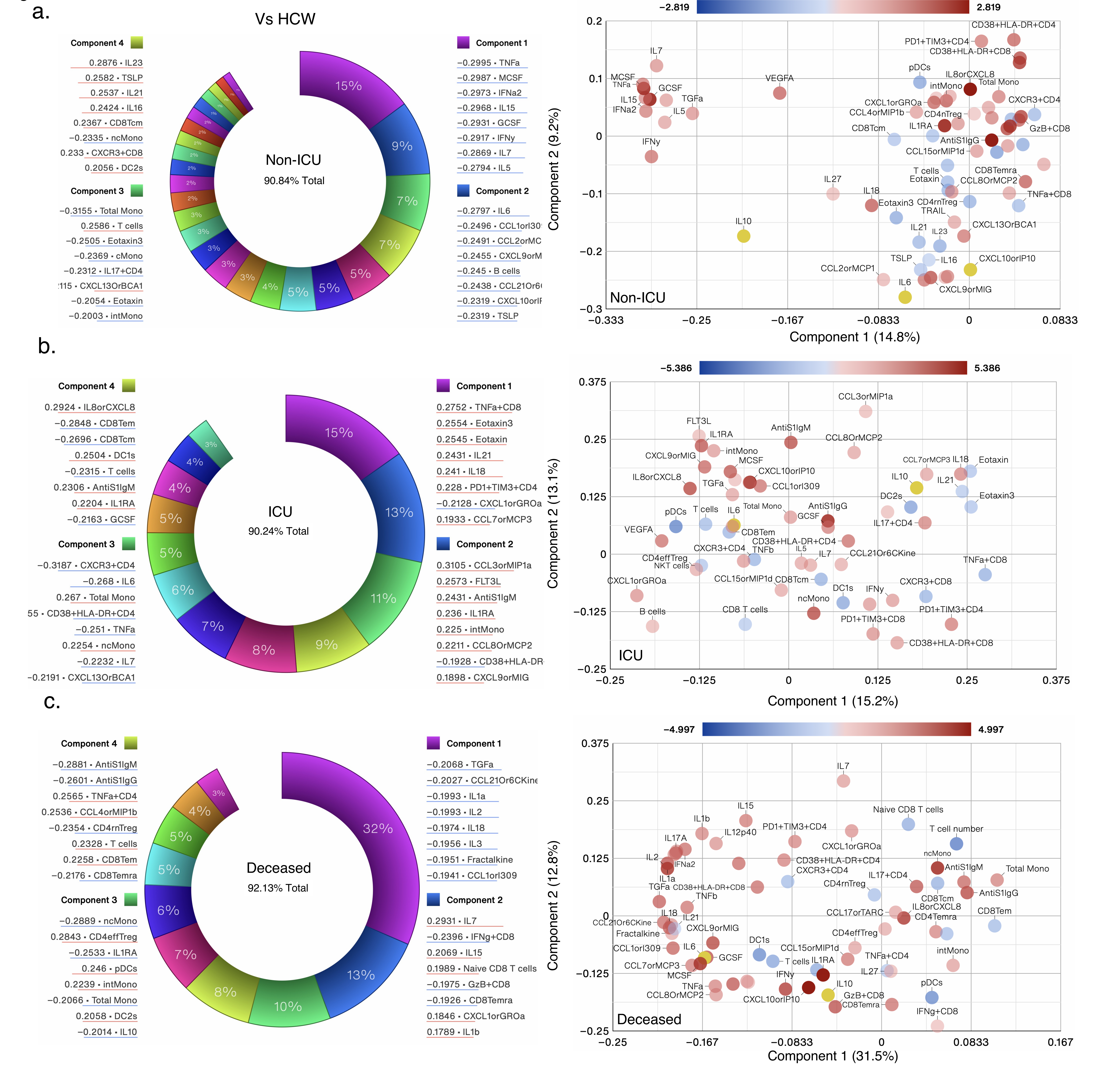
a.
Vs HCW
Component 4
0.2876 • IL23
0.2582 • TSLP
0.2537 • IL21
0.2424 • IL16
0.2367 • CD8Tcm
−0.2335 • ncMono
0.233 • CXCR3+CD8
0.2056 • DC2s
Component 3
−0.3155 • Total Mono
0.2586 • T cells
−0.2505 • Eotaxin3
−0.2369 • cMono
−0.2312 • IL17+CD4
−0.2054 • Eotaxin
−0.2003 • intMono
Non-ICU
90.84% Total
Component 1
−0.2995 • TNFa
−0.2987 • MCSF
−0.2973 • IFNa2
−0.2968 • IL15
−0.2931 • GCSF
−0.2917 • IFNy
−0.2869 • IL7
−0.2794 • IL5
Component 2
−0.2797 • IL6
−0.245 • B cells
−0.2319 • TSLP
−2.819
2.819
Non-ICU
Component 1 (14.8%)
Component 2 (9.2%)
b.
Component 4
0.2924 • IL8orCXCL8
−0.2848 • CD8Tem
−0.2696 • CD8Tcm
0.2504 • DC1s
−0.2315 • T cells
0.2306 • AntiS1IgM
0.2204 • IL1RA
−0.2163 • GCSF
Component 3
−0.3187 • CXCR3+CD4
−0.268 • IL6
0.267 • Total Mono
−0.251 • TNFa
0.2254 • ncMono
−0.2232 • IL7
−0.2191 • CXCL13OrBCA1
ICU
90.24% Total
Component 1
0.2752 • TNFa+CD8
0.2554 • Eotaxin3
0.2545 • Eotaxin
0.2431 • IL21
0.241 • IL18
0.228 • PD1+TIM3+CD4
−0.2128 • CXCL1orGROa
0.1933 • CCL7orMCP3
Component 2
0.3105 • CCL3orMIP1a
0.2573 • FLT3L
0.2431 • AntiS1IgM
0.236 • IL1RA
0.225 • intMono
0.2211 • CCL8OrMCP2
0.1898 • CXCL9orMIG
−5.386
5.386
ICU
Component 1 (15.2%)
Component 2 (13.1%)
c.
Component 4
−0.2881 • AntiS1IgM
−0.2601 • AntiS1IgG
0.2565 • TNFa+CD4
0.2536 • CCL4orMIP1b
−0.2354 • CD4rnTreg
0.2328 • T cells
0.2258 • CD8Tem
−0.2176 • CD8Temra
Component 3
−0.2889 • ncMono
0.2843 • CD4effTreg
−0.2533 • IL1RA
0.246 • pDCs
0.2239 • intMono
−0.2066 • Total Mono
0.2058 • DC2s
−0.2014 • IL10
Deceased
92.13% Total
Component 1
−0.2068 • TGFa
−0.2027 • CCL21Or6CKine
−0.1993 • IL1a
−0.1993 • IL2
−0.1974 • IL18
−0.1956 • IL3
−0.1951 • Fractalkine
−0.1941 • CCL1orl309
Component 2
0.2931 • IL7
−0.2396 • IFNg+CD8
0.2069 • IL15
0.1989 • Naive CD8 T cells
−0.1975 • GzB+CD8
−0.1926 • CD8Temra
0.1846 • CXCL1orGROa
0.1789 • IL1b
−4.997
4.997
Deceased
Component 1 (31.5%)
Component 2 (12.8%)

Fig. S3

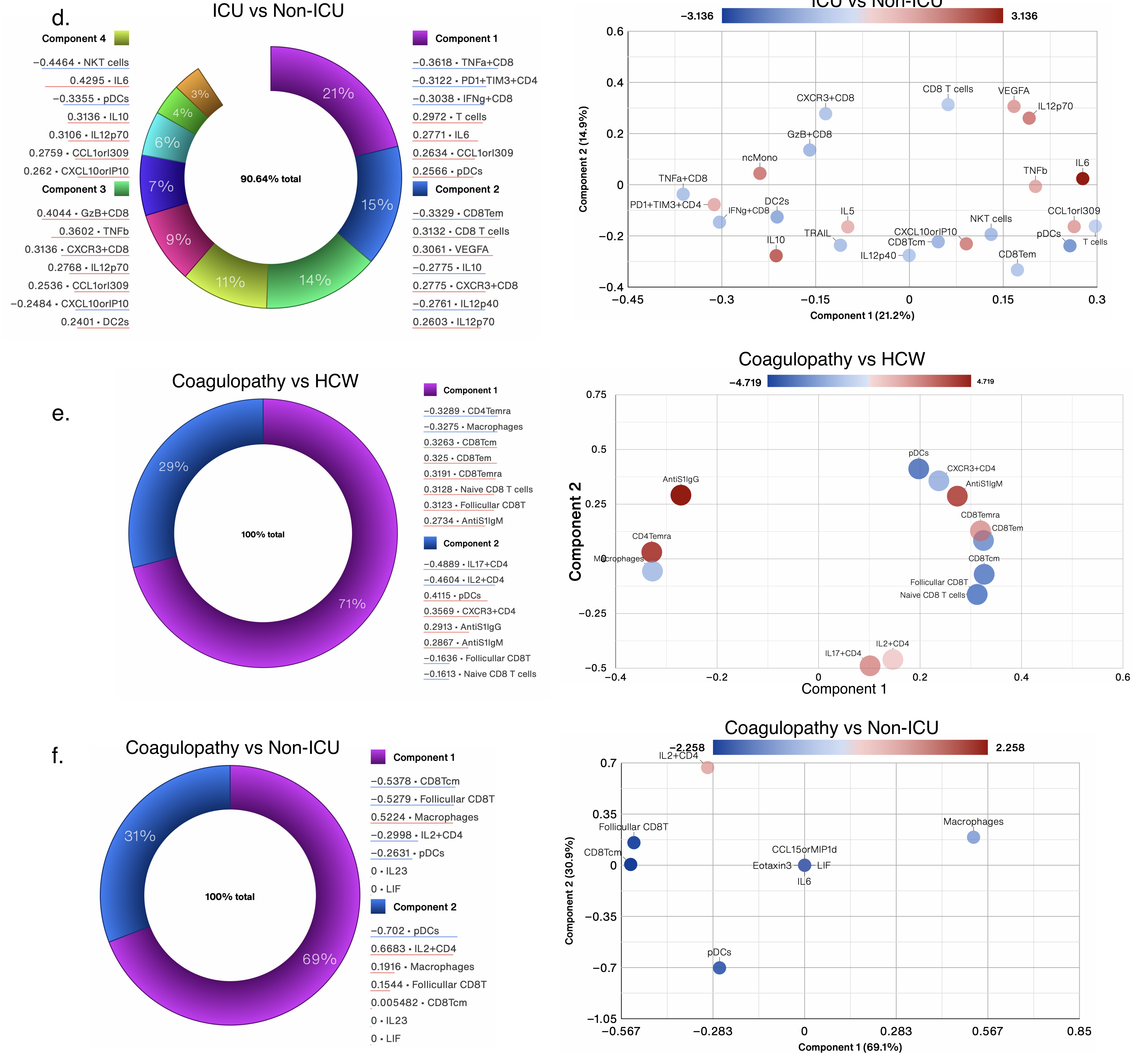

d.
ICU vs Non-ICU
Component 4
−0.4464 • NKT cells
0.4295 • IL6
−0.3355 • pDCs
0.3136 • IL10
0.3106 • IL12p70
0.2759 • CCL1orl309
0.262 • CXCL10orIP10
Component 3
0.4044 • GzB+CD8
0.3602 • TNFb
0.3136 • CXCR3+CD8
0.2768 • IL12p70
0.2536 • CCL1orl309
−0.2484 • CXCL10orIP10
0.2401 • DC2s
3%
4%
6%
7%
9%
11%
14%
15%
21%
90.64% total
Component 1
−0.3618 • TNFa+CD8
−0.3122 • PD1+TIM3+CD4
−0.3038 • IFNg+CD8
0.2972 • T cells
0.2771 • IL6
0.2634 • CCL1orl309
0.2566 • pDCs
Component 2
−0.3329 • CD8Tem
0.3132 • CD8 T cells
0.3061 • VEGFA
−0.2775 • IL10
0.2775 • CXCR3+CD8
−0.2761 • IL12p40
0.2603 • IL12p70
ICU vs Non-ICU
−3.136
3.136
Component 2 (14.9%)
Component 1 (21.2%)
0.6
0.4
0.2
0
−0.2
−0.4
−0.45
−0.3
−0.15
0
0.15
0.3
CD8 T cells
VEGFA
IL12p70
CXCR3+CD8
GzB+CD8
ncMono
IL6
TNFb
TNFa+CD8
PD1+TIM3+CD4
IFNg+CD8
DC2s
IL5
NKT cells
CCL1orl309
pDCs
T cells
IL10
TRAIL
CXCL10orIP10
CD8Tcm
IL12p40
CD8Tem
e.
Coagulopathy vs HCW
29%
71%
100% total
Component 1
−0.3289 • CD4Temra
−0.3275 • Macrophages
0.3263 • CD8Tcm
0.325 • CD8Tem
0.3191 • CD8Temra
0.3128 • Naive CD8 T cells
0.3123 • Follicullar CD8T
0.2734 • AntiS1IgM
Component 2
−0.4889 • IL17+CD4
−0.4604 • IL2+CD4
0.4115 • pDCs
0.3569 • CXCR3+CD4
0.2913 • AntiS1IgG
0.2867 • AntiS1IgM
−0.1636 • Follicullar CD8T
−0.1613 • Naive CD8 T cells
Coagulopathy vs HCW
−4.719
4.719
Component 2
Component 1
0.75
0.5
0.25
0
−0.25
−0.5
−0.4
−0.2
0
0.2
0.4
0.6
pDCs
CXCR3+CD4
AntiS1IgM
AntiS1IgG
CD8Temra
CD8Tem
CD4Temra
Macrophages
CD8Tcm
Follicullar CD8T
Naive CD8 T cells
IL2+CD4
IL17+CD4
f.
Coagulopathy vs Non-ICU
31%
69%
100% total
Component 1
−0.5378 • CD8Tcm
−0.5279 • Follicullar CD8T
0.5224 • Macrophages
−0.2998 • IL2+CD4
−0.2631 • pDCs
0 • IL23
0 • LIF
Component 2
−0.702 • pDCs
0.6683 • IL2+CD4
0.1916 • Macrophages
0.1544 • Follicullar CD8T
0.005482 • CD8Tcm
0 • IL23
0 • LIF
Coagulopathy vs Non-ICU
−2.258
2.258
Component 2 (30.9%)
Component 1 (69.1%)
0.7
0.35
0
−0.35
−0.7
−1.05
−0.567
−0.283
0
0.283
0.567
0.85
IL2+CD4
Follicullar CD8T
Macrophages
CD8Tcm
CCL15orMIP1d
Eotaxin3
LIF
IL6
pDCs

Fig.S4

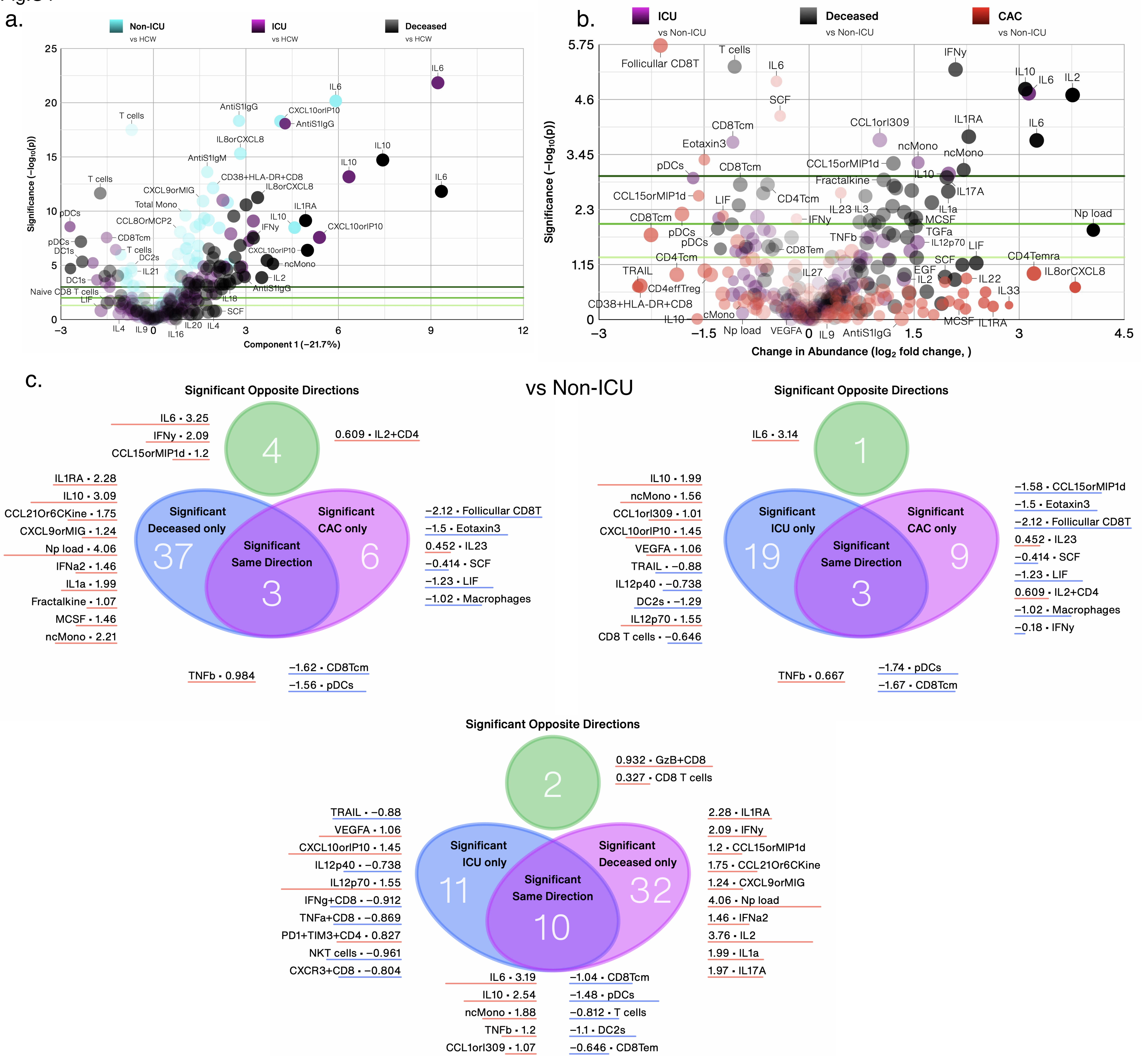
a.
Non-ICU vs HCW
ICU vs HCW
Deceased vs HCW
Significance (−log10(p))
Component 1 (−21.7%)
IL6
AntiS1IgG
CXCL10orIP10
T cells
IL8orCXCL8
IL10
AntiS1IgM
CD38+HLA-DR+CD8
CXCL9orMIG
Total Mono
IL1RA
CCL8OrMCP2
CD8Tcm
IFNy
pDCs
DC1s
DC2s
IL21
ncMono
IL2
Naive CD8 T cells
LIF
IL18
SCF
IL4
IL9
IL16
IL20
b.
ICU vs Non-ICU
Deceased vs Non-ICU
CAC vs Non-ICU
Significance (−log10(p))
Change in Abundance (log2 fold change,)
Follicular CD8T
T cells
IFNy
IL2
IL1RA
CCL1orI309
ncMono
Eotaxin3
CCL15orMIP1d
Fractalkine
IL17A
CD4Tcm
IL23
IL3
MCSF
Np load
TGFa
TNFb
IL12p70
CD8Tem
CD4Temra
CD4effTreg
CD38+HLA-DR+CD8
TRAIL
IL27
EGF
IL22
IL33
cMono
Np load
VEGFA
AntiS1IgG
c.
vs Non-ICU
Significant Opposite Directions
4
IL6 • 3.25
IFNy • 2.09
CCL15orMIP1d • 1.2
0.609 • IL2+CD4
Significant Deceased only
37
Significant Same Direction
3
Significant CAC only
6
IL1RA • 2.28
IL10 • 3.09
CCL21Or6CKine • 1.75
CXCL9orMIG • 1.24
Np load • 4.06
IFNa2 • 1.46
IL1a • 1.99
Fractalkine • 1.07
MCSF • 1.46
ncMono • 2.21
−2.12 • Follicular CD8T
−1.5 • Eotaxin3
0.452 • IL23
−0.414 • SCF
−1.23 • LIF
−1.02 • Macrophages
TNFb • 0.984
−1.62 • CD8Tcm
−1.56 • pDCs
Significant Opposite Directions
1
IL6 • 3.14
Significant ICU only
19
Significant Same Direction
3
Significant CAC only
9
IL10 • 1.99
ncMono • 1.56
CCL1orI309 • 1.01
CXCL10orIP10 • 1.45
VEGFA • 1.06
TRAIL • −0.88
IL12p40 • −0.738
DC2s • −1.29
IL12p70 • 1.55
CD8 T cells • −0.646
−1.58 • CCL15orMIP1d
−1.5 • Eotaxin3
−2.12 • Follicular CD8T
0.452 • IL23
−0.414 • SCF
−1.23 • LIF
0.609 • IL2+CD4
−1.02 • Macrophages
−0.18 • IFNy
TNFb • 0.667
−1.74 • pDCs
−1.67 • CD8Tcm
Significant Opposite Directions
2
0.932 • GzB+CD8
0.327 • CD8 T cells
Significant ICU only
11
Significant Same Direction
10
Significant Deceased only
32
TRAIL • −0.88
VEGFA • 1.06
CXCL10orIP10 • 1.45
IL12p40 • −0.738
IL12p70 • 1.55
IFNg+CD8 • −0.912
TNFa+CD8 • −0.869
PD1+TIM3+CD4 • 0.827
NKT cells • −0.961
CXCR3+CD8 • −0.804
2.28 • IL1RA
2.09 • IFNy
1.2 • CCL15orMIP1d
1.75 • CCL21Or6CKine
1.24 • CXCL9orMIG
4.06 • Np load
1.46 • IFNa2
3.76 • IL2
1.99 • IL1a
1.97 • IL17A
IL6 • 3.19
IL10 • 2.54
ncMono • 1.88
TNFb • 1.2
CCL1orI309 • 1.07
−1.04 • CD8Tcm
−1.48 • pDCs
−0.812 • T cells
−1.1 • DC2s
−0.646 • CD8Tem

Fig. S5

a.

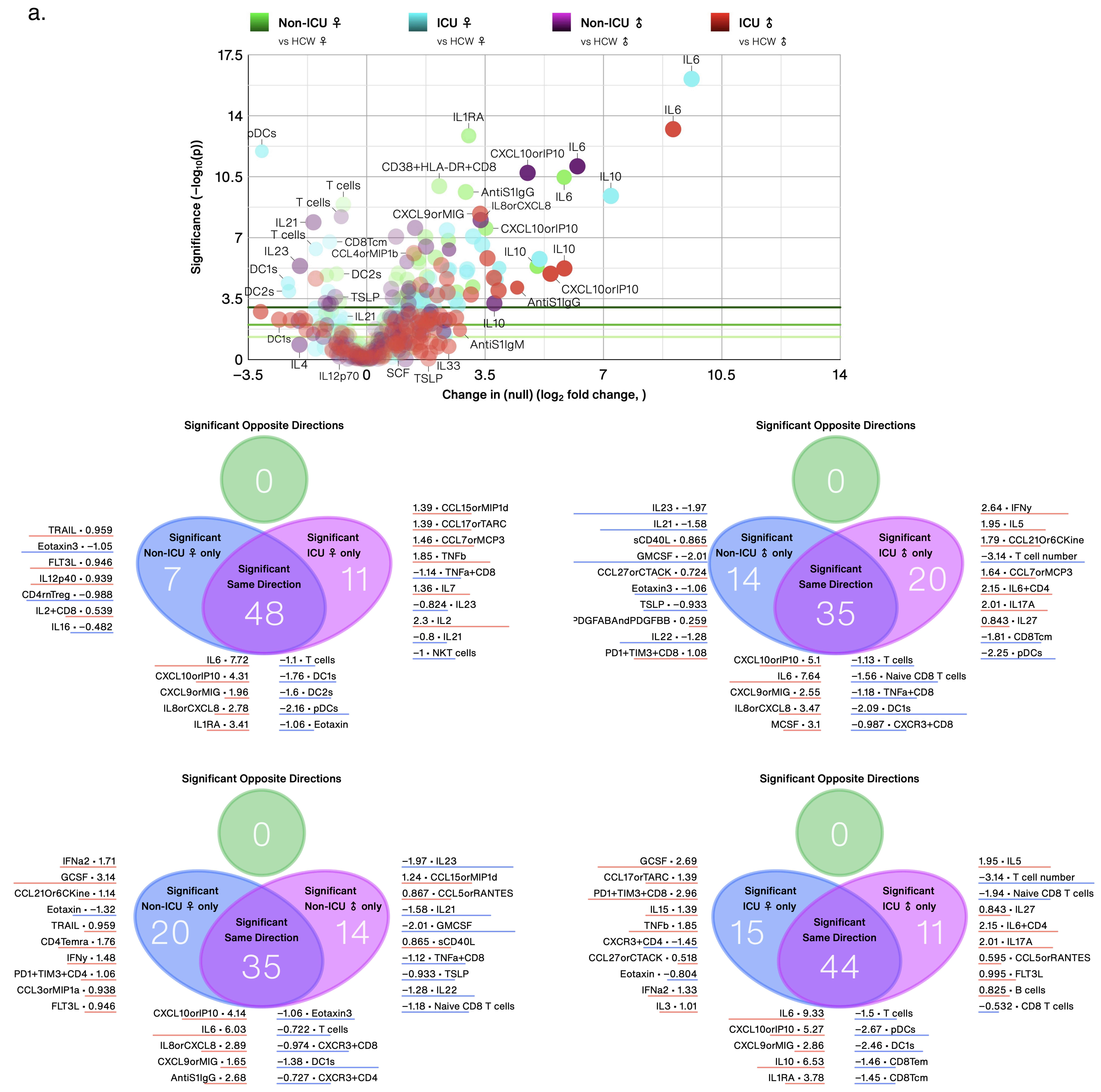

Non-ICU ♀ vs HCW ♀
ICU ♀ vs HCW ♀
Non-ICU ♂ vs HCW ♂
ICU ♂ vs HCW ♂
Significance (−log10(p))
Change in (null) (log2 fold change,)
IL6
IL1RA
pDCs
CXCL10orIP10
CD38+HLA-DR+CD8
AntiS1IgG
IL8orCXCL8
CXCL9orMIG
IL10
T cells
IL21
IL23
CD8Tcm
CCL4orMIP1b
DC1s
DC2s
TSLP
AntiS1IgM
IL4
IL12p70
SCF
IL33
Significant Opposite Directions
0
Significant Non-ICU ♀ only
7
Significant ICU ♀ only
11
Significant Same Direction
48
TRAIL • 0.959
Eotaxin3 • −1.05
FLT3L • 0.946
IL12p40 • 0.939
CD4rnTreg • −0.988
IL2+CD8 • 0.539
IL16 • −0.482
1.39 • CCL15orMIP1d
1.39 • CCL17orTARC
1.46 • CCL7orMCP3
1.85 • TNFb
−1.14 • TNFa+CD8
1.36 • IL7
−0.824 • IL23
2.3 • IL2
−0.8 • IL21
−1 • NKT cells
IL6 • 7.72
CXCL10orIP10 • 4.31
CXCL9orMIG • 1.96
IL8orCXCL8 • 2.78
IL1RA • 3.41
−1.1 • T cells
−1.76 • DC1s
−1.6 • DC2s
−2.16 • pDCs
−1.06 • Eotaxin
Significant Opposite Directions
0
Significant Non-ICU ♂ only
14
Significant ICU ♂ only
20
Significant Same Direction
35
IL23 • −1.97
IL21 • −1.58
sCD40L • 0.865
GMCSF • −2.01
CCL27orCTACK • 0.724
Eotaxin3 • −1.06
TSLP • −0.933
PDGFABAndPDGFBB • 0.259
IL22 • −1.28
PD1+TIM3+CD8 • 1.08
2.64 • IFNy
1.95 • IL5
1.79 • CCL21Or6CKine
−3.14 • T cell number
1.64 • CCL7orMCP3
2.15 • IL6+CD4
2.01 • IL17A
0.843 • IL27
−1.81 • CD8Tcm
−2.25 • pDCs
CXCL10orIP10 • 5.1
IL6 • 7.64
CXCL9orMIG • 2.55
IL8orCXCL8 • 3.47
MCSF • 3.1
−1.13 • T cells
−1.56 • Naive CD8 T cells
−1.18 • TNFa+CD8
−2.09 • DC1s
−0.987 • CXCR3+CD8
Significant Opposite Directions
0
Significant Non-ICU ♀ only
20
Significant Non-ICU ♂ only
14
Significant Same Direction
35
IFNa2 • 1.71
GCSF • 3.14
CCL21Or6CKine • 1.14
Eotaxin • −1.32
TRAIL • 0.959
CD4Temra • 1.76
IFNy • 1.48
PD1+TIM3+CD4 • 1.06
CCL3orMIP1a • 0.938
FLT3L • 0.946
−1.97 • IL23
1.24 • CCL15orMIP1d
0.867 • CCL5orRANTES
−1.58 • IL21
−2.01 • GMCSF
0.865 • sCD40L
−1.12 • TNFa+CD8
−0.933 • TSLP
−1.28 • IL22
−1.18 • Naive CD8 T cells
CXCL10orIP10 • 4.14
IL6 • 6.03
IL8orCXCL8 • 2.89
CXCL9orMIG • 1.65
AntiS1IgG • 2.68
−1.06 • Eotaxin3
−0.722 • T cells
−0.974 • CXCR3+CD8
−1.38 • DC1s
−0.727 • CXCR3+CD4
Significant Opposite Directions
0
Significant ICU ♀ only
15
Significant ICU ♂ only
11
Significant Same Direction
44
GCSF • 2.69
CCL17orTARC • 1.39
PD1+TIM3+CD8 • 2.96
IL15 • 1.39
TNFb • 1.85
CXCR3+CD4 • −1.45
CCL27orCTACK • 0.518
Eotaxin • −0.804
IFNa2 • 1.33
IL3 • 1.01
1.95 • IL5
−3.14 • T cell number
−1.94 • Naive CD8 T cells
0.843 • IL27
2.15 • IL6+CD4
2.01 • IL17A
0.595 • CCL5orRANTES
0.995 • FLT3L
0.825 • B cells
−0.532 • CD8 T cells
IL6 • 9.33
CXCL10orIP10 • 5.27
CXCL9orMIG • 2.86
IL10 • 6.53
IL1RA • 3.78
−1.5 • T cells
−2.67 • pDCs
−2.46 • DC1s
−1.46 • CD8Tem
−1.45 • CD8Tcm

Fig. S5

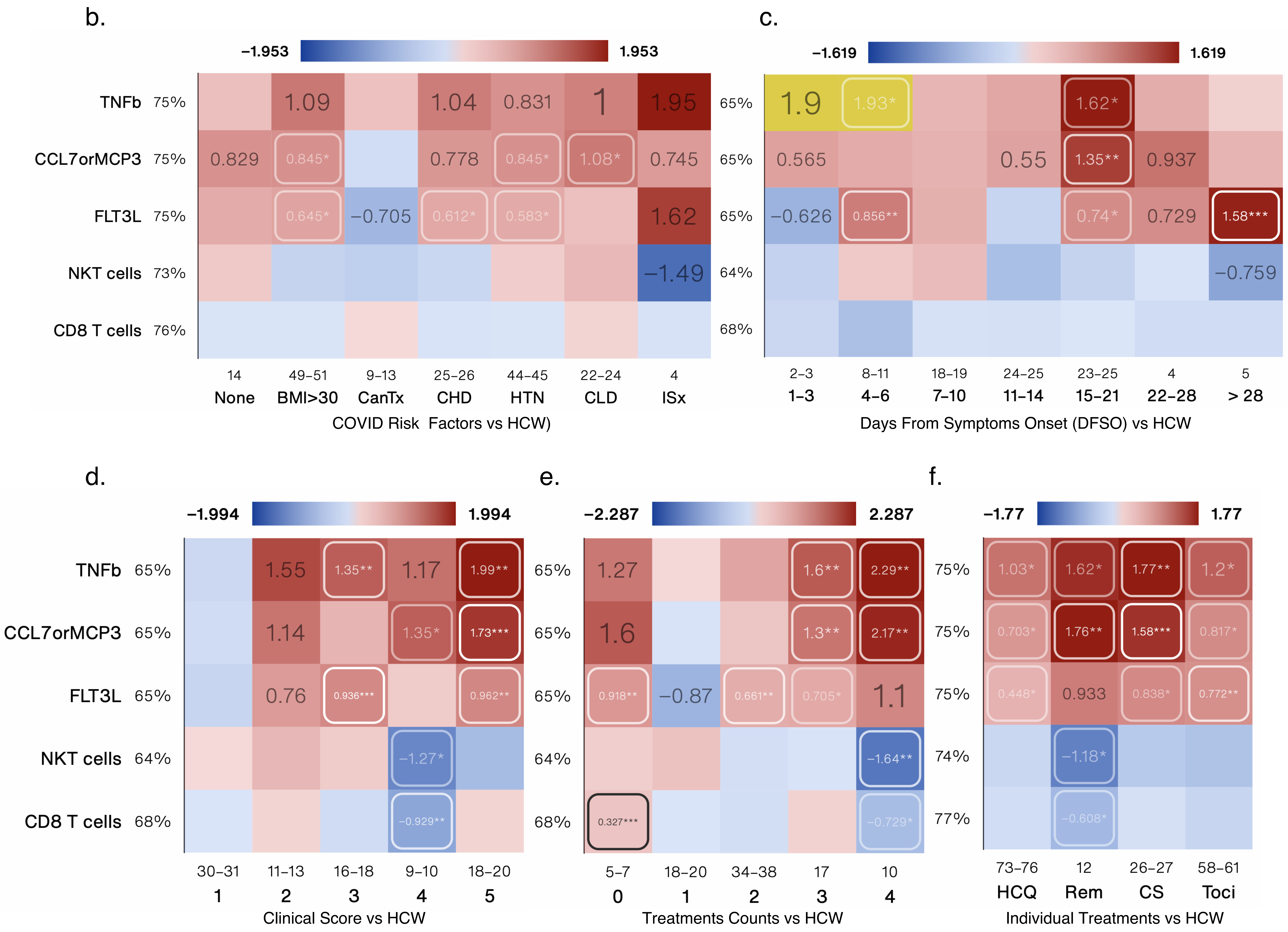
b.
−1.953
1.953
TNFb 75%
CCL7orMCP3 75%
FLT3L 75%
NKT cells 73%
CD8 T cells 76%
1.09
1.04
0.831
1
1.95
0.829
0.845*
0.778
0.845*
1.08*
0.745
0.645*
−0.705
0.612*
0.583*
1.62
−1.49
14
None
49–51
BMI>30
9–13
CanTx
25–26
CHD
44–45
HTN
22–24
CLD
4
ISx
COVID Risk Factors vs HCW)
c.
−1.619
1.619
65%
65%
65%
64%
68%
1.9
1.93*
1.62*
0.565
0.55
1.35**
0.937
−0.626
0.856**
0.74*
0.729
1.58***
−0.759
2–3
1–3
8–11
4–6
18–19
7–10
24–25
11–14
23–25
15–21
4
22–28
5
> 28
Days From Symptoms Onset (DFSO) vs HCW
d.
−1.994
1.994
TNFb 65%
CCL7orMCP3 65%
FLT3L 65%
NKT cells 64%
CD8 T cells 68%
1.55
1.35**
1.17
1.99**
1.14
1.35*
1.73***
0.76
0.936***
0.962**
−1.27*
−0.929**
30–31
1
11–13
2
16–18
3
9–10
4
18–20
5
Clinical Score vs HCW
e.
−2.287
2.287
65%
65%
65%
64%
68%
1.27
1.6**
2.29**
1.6
1.3**
2.17**
0.918**
−0.87
0.661**
0.705*
1.1
−1.64**
0.327***
−0.729*
5–7
0
18–20
1
34–38
2
17
3
10
4
Treatments Counts vs HCW
f.
−1.77
1.77
75%
75%
75%
74%
77%
1.03*
1.62*
1.77**
1.2*
0.703*
1.76**
1.58***
0.817*
0.448*
0.933
0.838*
0.772**
−1.18*
−0.608*
73–76
HCQ
12
Rem
26–27
CS
58–61
Toci
Individual Treatments vs HCW

Fig.S6

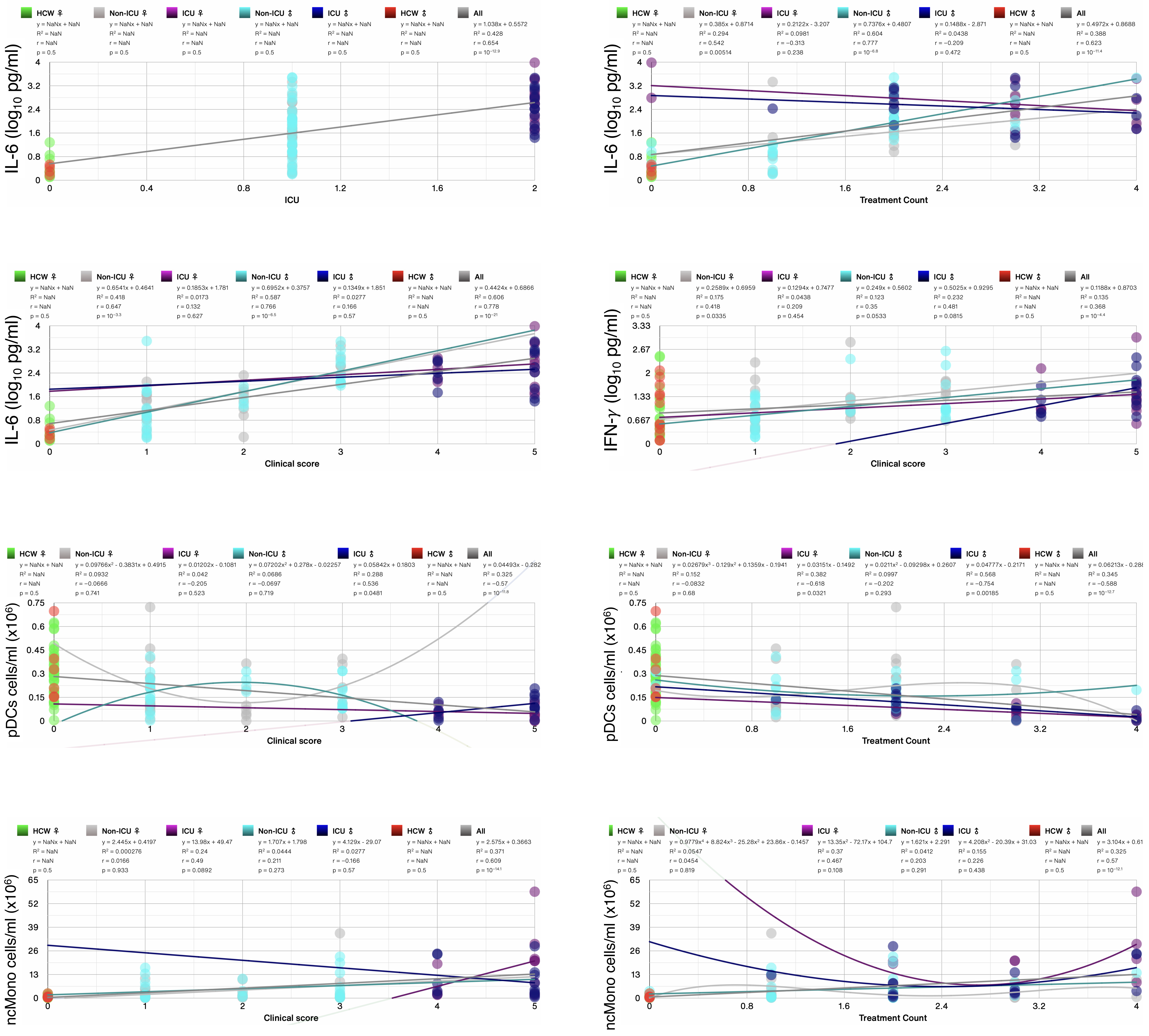

HCW ♀
Non-ICU ♀
ICU ♀
Non-ICU ♂
ICU ♂
HCW ♂
All
y = 1.038x + 0.5572
R² = 0.428
r = 0.654
p = 10^-12.9
IL-6 (log10 pg/ml)
ICU
y = 0.385x + 0.8714
R² = 0.294
r = 0.542
p = 0.00514
y = 0.2122x - 3.207
R² = 0.0981
r = −0.313
p = 0.238
y = 0.7376x + 0.4807
R² = 0.604
r = 0.777
p = 10^-6.8
y = 0.1488x - 2.871
R² = 0.0438
r = −0.209
p = 0.472
y = 0.4972x + 0.8688
R² = 0.388
r = 0.623
p = 10^-11.4
Treatment Count
y = 0.6541x + 0.4641
R² = 0.418
r = 0.647
p = 10^-3.3
y = 0.1853x + 1.781
R² = 0.0173
r = 0.132
p = 0.627
y = 0.6952x + 0.3757
R² = 0.587
r = 0.766
p = 10^-6.5
y = 0.1349x + 1.851
R² = 0.0277
r = 0.166
p = 0.57
y = 0.4424x + 0.6866
R² = 0.606
r = 0.778
p = 10^-21
Clinical score
IFN-γ (log10 pg/ml)
y = 0.2589x + 0.6959
R² = 0.175
r = 0.418
p = 0.0335
y = 0.1294x + 0.7477
R² = 0.0438
r = 0.209
p = 0.454
y = 0.249x + 0.5602
R² = 0.123
r = 0.35
p = 0.0533
y = 0.5025x + 0.9295
R² = 0.232
r = 0.481
p = 0.0815
y = 0.1188x + 0.8703
R² = 0.135
r = 0.368
p = 10^-4.4
pDCs cells/ml (x10^6)
y = 0.09766x² - 0.3831x + 0.4915
R² = 0.0932
r = −0.0666
p = 0.741
y = 0.01202x - 0.1081
R² = 0.042
r = −0.205
p = 0.523
y = 0.07202x² + 0.278x - 0.02257
R² = 0.0686
r = −0.0697
p = 0.719
y = 0.05842x + 0.1803
R² = 0.288
r = 0.536
p = 0.0481
y = 0.04493x - 0.282
R² = 0.325
r = −0.57
p = 10^-11.8
y = 0.02679x³ - 0.129x² + 0.1359x - 0.1941
R² = 0.152
r = −0.0832
p = 0.68
y = 0.03151x - 0.1492
R² = 0.382
r = −0.618
p = 0.0321
y = 0.0211x² - 0.09298x + 0.2607
R² = 0.0997
r = −0.202
p = 0.293
y = 0.04777x - 0.2171
R² = 0.568
r = −0.754
p = 0.00185
y = 0.06213x - 0.288
R² = 0.345
r = −0.588
p = 10^-12.7
ncMono cells/ml (x10^6)
y = 2.445x + 0.4197
R² = 0.000276
r = 0.0166
p = 0.933
y = 13.98x + 49.47
R² = 0.24
r = 0.49
p = 0.0892
y = 1.707x + 1.798
R² = 0.0444
r = 0.211
p = 0.273
y = 4.129x - 29.07
R² = 0.0277
r = −0.166
p = 0.57
y = 2.575x + 0.3663
R² = 0.371
r = 0.609
p = 10^-14.1
y = 0.9779x⁴ + 8.824x³ - 25.28x² + 23.86x - 0.1457
R² = 0.0547
r = 0.0454
p = 0.819
y = 13.35x² - 72.17x + 104.7
R² = 0.37
r = 0.467
p = 0.108
y = 1.621x + 2.291
R² = 0.0412
r = 0.203
p = 0.291
y = 4.208x² - 20.39x + 31.03
R² = 0.155
r = 0.226
p = 0.438
y = 3.104x + 0.61
R² = 0.325
r = 0.57
p = 10^-12.1
