# Supplementary material for: Immunological Misfiring and Sex Differences/Similarities in Early COVID-19 Studies: Missed Opportunities of Making a Real IMPACT
Source: Cells. 2023 Nov 8;12(22):2591. doi: 10.3390/cells12222591 (PMC10670326; doi:10.3390/cells12222591)
Supplement: Supplementary file 1 [file cells-12-02591-s001.zip › cells-2638127-supplementary/SupplemetaryDataCells-Revised/ImpactAB_JK-Supplementary FigureS1-5andTableS1-5_LegendsOct31.pdf]

Title: Immunological misfiring and sex differences/similarities in early COVID-19 studies: missed opportunities of making a real IMPACT

Aditi Bhargava[1, 2,*], Johannes D Knapp[2]

[1] Affiliation 1 Center for Reproductive Sciences and Department of ObGyn, University of California San Francisco, San Francisco, CA 94143; aditi.bhargava@ucsf.edu
[2] Affiliation 2: Aseesa Inc., CA, USA; jk@aseesa.com
* Correspondence: Aditi.bhargava@ucsf.edu

Supplementary Figures S1-6. Changed immunological and biological measures in COVID-19 patients.

Figure S1. Scatter plots showing the four measures most significantly correlated with SARS-CoV-2 (**a**) Saliva load, and (**b**) Np Load; the goodness-of-fit (R^2), Pearson's r, and p-values for each test group are shown. Not a Number (NaN) indicates that samples in this group did not contain any values. N/group: HCW: 114; Non-ICU: 60; ICU: 17; Deceased: 16.

Figure S2. Scatter plots (left) and bar charts (right) for cytokines that exhibited stronger correlations in females than in males, including between IFNα2 and IFNγ, for CCL21 with CCL1 and fractalkine, and for CCL8 with CCL1. Goodness-of-fit (R^2), Person's r, and p-values shown for each group. CCL1, CCL21, and CXCL10 appear in several correlations between different measures in this cohort and may be significant for predicting disease progression. Number next to the groups denote actual numbers of patients in which each measure was detected. N/group: HCW♀: 87; Non-ICU♀: 30; ICU♀: 16; Non-ICU♂: 33; ICU♂: 14; HCW♂: 27.

Figure S3. PCAs for significantly changed symbols in non-ICU **(a)**, ICU **(b)**, deceased **(c)** and coagulopathy-affected **(e)** COVID-19 patients versus HCW healthy controls, and in ICU **(d)**, and coagulopathy-affected **(f)** COVID-19 patients versus non-ICU patients: donut plots showing the primary components necessary to explain at least 90% of the group's variance, and the 9 symbols most correlated with each of the first four primary components (left), and PCA biplots for the first two components (right), with the color of points denoting $\log_2$ fold change versus the respective control group.

Figure S4. Volcano plots for **(a)** non-ICU, ICU, and deceased COVID-19 patients versus HCW healthy controls, and for **(b)** ICU, deceased and coagulopathy-affected (CAC) COVID-19 patients versus non-ICU patients. **(c)** Venn diagram contrasting significantly changed measures between subpopulations of ICU patients compared to non-ICU patients.

Figure S5. Volcano plots for **(a)** non-ICU and ICU amle and female COVID-19 patients versus non-ICU patients. Venn diagram contrasting significantly changed measures between male and female ICU patients and non-ICU patients compared with HCW healthy controls. Heat maps showing the measures from (**b-f**) by patient status and sex, and by DFSO, clinical score, COVID-19 risk factors, treatment and treatment count. Numbers below denote the number of data points in that group (n). All values shows are average $\log_2$ fold change (untransformed) versus the respective control group average, outlier-adjusted. Value labels were drawn for values greater than 33% of the heat map's maximum value. The

maximum value does not include outliers (highlighted in yellow) defined as less than Q1 − 1.5(IQR) or greater than Q3 + 1.5(IQR). Labels for values less than 16.67% of the maximum are drawn in black for legibility. *, ** and *** denote $p < 0.05$, 0.01 and 0.001 by Welch's t-test. Cancer treatment (Tx) received in prior 1 year; CHD: chronic heart diseases; HTN: hypertension; CLD: chronic lung diseases; ISx: immunosuppressed patients. HQ: hydroxychloroquine; Remdes: Remdesivir; Cort: high dose of corticosteroid; Toci: Tocilizumab. V/A: adjusted value-to-average. IQR: Interquartile range.

Figure S6. Scatter plots showing correlations for IL-6 with ICU admission, treatment count and clinical score; plasmacytoid dendritic cells (pDCs) with clinical score and treatment count, and nonclassical monocytes (ncMono) with clinical score and treatment count, with goodness-of-fit (R^2), Pearson's r, and p-values shown for each group.

Supplementary Tables S1-4 Legends:

Table S1. SARS-CoV-2 viral load in nasopharyngeal (Np) and saliva samples in IMPACT Cohort patients. Breakdown of a total of 179* data points from a total of 98 patients. Two or more longitudinal data points were collected from a total of 59 patients that were hospitalized in Yale between March and May of 2020. *: DFSO for a few patients were missing/not reported and hence those patients were not included in our analysis for DFSO. Numbers in parenthesis (0) indicates patients with confirmed 0 values for viral load in both saliva and Np samples. Missing values represent samples was not collected and/or tested. The numbers of datapoints/patients shown in the middle column were used in our reanalysis.

Table S2. All Characteristics. All biological and clinical measures' correlations of COVID-19$^+$ patients (sex aggregated) with each other using heath care workers (HCW) as comparison group.

Table S3. All significantly changed ($p<0.05$) biological and clinical measures in COVID-19$^+$ patients (sex aggregated) versus heath care workers (HCW) as comparison group. Most significantly changed measures are shown in descending order.

Table S4. All significantly changed ($p<0.05$) biological and clinical measures in COVID-19$^+$ non-ICU patients (sex aggregated) versus heath care workers (HCW) as comparison group. Most significantly changed measures are shown in descending order.

Table S5. All significantly changed ($p<0.05$) biological and clinical measures in COVID-19$^+$ ICU patients (sex aggregated) versus heath care workers (HCW) as comparison group. Most significantly changed measures are shown in descending order.
