# Supplementary material for: Immunological Misfiring and Sex Differences/Similarities in Early COVID-19 Studies: Missed Opportunities of Making a Real IMPACT
Source: Cells. 2023 Nov 8;12(22):2591. doi: 10.3390/cells12222591 (PMC10670326; doi:10.3390/cells12222591)
Supplement: Supplementary file 1 [file cells-12-02591-s001.zip › cells-2638127-supplementary/SupplemetaryDataCells-Revised/TableS1-Cells.pdf]

Table S1. SARS-CoV-2 viral load in nasopharyngeal (Np) and saliva samples in IMPACT Cohort patients.

Breakdown of a total of 179* data points from a total of 98 patients. Two or more longitudinal data points were collected from a total of 59 patients that were hospitalized in Yale between March and May of 2020. *: DFSO for a few patients were missing/not reported and hence those patients were not included in our analysis for DFSO. Numbers in parenthesis (0) indicates patients with confirmed 0 values for viral load in both saliva and Np samples. Missing values represent samples was not collected and/or tested. The numbers in the middle column is the actual patients used in our reanalysis.

DFSO	Patient Data Points			S or Nps> 0			S or Nps = Missing/0		
	Both	Female	Male	Both	Female	Male	Both	Female	Male
1-3	6	5	1	3	2	1	3 (2)	3 (2)	0 (0)
4-6	14	7	7	11	5	6	3 (0)	2 (0)	1 (0)
7-10	37	18	19	19	8	11	16 (8)	10 (6)	6 (2)
11-14	44	21	23	26	12	14	18 (8)	7 (2)	11 (6)
15-21	44	27	17	25	16	9	18 (6)	13 (3)	5 (3)
22-27	19	11	8	4	1	3	18 (3)	10 (1)	8 (2)
⩾ 28	15	7	8	5	2	3	10 (2)	5 (1)	5 (1)
TOTAL	179	96	83	93	46	47	86 (29)	50 (15)	36 (14)

	Clinical Score			S or Nps > 0			S or Nps = 0		
	Both	Female	Male	Both	Female	Male	Both	Female	Male
1	64	37	27	32	15	17	10	6	4
2	19	12	7	13	9	4	4	2	2
3	49	22	27	18	6	12	11	5	6
4	17	11	6	10	5	5	1	1	0
5	29	13	16	20	11	9	3	1	2
TOTAL									
